# Impact of a Novel Smartphone App (CureApp Smoking Cessation) on Nicotine Dependence: Prospective Single-Arm Interventional Pilot Study

**DOI:** 10.2196/12694

**Published:** 2019-02-19

**Authors:** Katsunori Masaki, Hiroki Tateno, Naofumi Kameyama, Eriko Morino, Riri Watanabe, Kazuma Sekine, Tomohiro Ono, Kohta Satake, Shin Suzuki, Akihiro Nomura, Tomoko Betsuyaku, Koichi Fukunaga

**Affiliations:** 1 Division of Pulmonary Medicine, Department of Medicine Keio University School of Medicine Tokyo Japan; 2 Department of Internal Medicine Saitama City Hospital Saitama Japan; 3 Department of Respiratory Medicine, Disease Control and Prevention Center National Center for Global Health and Medicine Tokyo Japan; 4 Department of General Internal Medicine National Center for Global Health and Medicine Tokyo Japan; 5 Akao Clinic Yokohama Japan; 6 Department of Respiratory Medicine Nippon Koukan Hospital Kawasaki Japan; 7 Ono Clinic Kakegawa Japan; 8 CureApp Institute Karuiwaza Japan; 9 CureApp Inc Tokyo Japan; 10 Innovative Clinical Research Center Kanazawa University Kanazawa Japan; 11 Department of Cardiovascular and Internal Medicine Kanazawa University Graduate School of Medicine Kanazawa Japan

**Keywords:** digital therapeutics, nicotine dependence, smoking cessation, smartphone application, telemedicine

## Abstract

**Background:**

Mobile apps have been considered to provide active and continuous support for smoking cessation. However, it is yet to be known whether a smoking cessation smartphone app improves long-term abstinence rates in nicotine-dependent patients.

**Objective:**

This study aimed to evaluate the long-term abstinence effect of a novel smartphone app, CureApp Smoking Cessation (CASC), in patients with nicotine dependence.

**Methods:**

In this prospective, interventional, multicenter, single-arm study, we provided the CASC app to all the participants, who used it daily for 24 weeks. The CASC app includes features to maximize the therapeutic effect of pharmacological therapies and counseling at outpatient clinics for smoking cessation. The primary endpoint was a continuous abstinence rate (CAR) from weeks 9 to 24, whereas secondary endpoints were CARs from weeks 9 to 12 and 9 to 52.

**Results:**

Of the 56 adult smokers recruited, 1 did not download the app; therefore, 55 participants constituted the full analysis sample. The CAR from weeks 9 to 24 was 64% (35/55, 95% CI 51%-76%), whereas the CARs from weeks 9 to 12 and 9 to 52 were 76% (42/55, 95% CI 65%-88%) and 58% (32/55, 95% CI 46%-71%), respectively. These CARs were better than the results of the national survey on outpatient clinics with regard to smoking cessation under the National Health Insurance Program and that of the varenicline phase 3 trial in Japan and the United States. There was only 1 participant who dropped out during the 12 weeks of the treatment period. This treatment decreased the scores related to withdrawal and craving symptoms.

**Conclusions:**

The addition of CASC to usual smoking cessation therapies resulted in high CARs, high patient retention rates, and improvement of cessation-related symptoms. The smartphone app CASC is a feasible and useful tool to help long-term continuous abstinence that can be combined with a standard smoking cessation treatment program.

## Introduction

### Background: Smoking Cessation Therapy

Smoking is a risk factor for cancer, respiratory disease, heart disease, and cerebral vascular disease [1]. Quitting smoking lowers the risk of smoking-related illnesses and can add years to the lives of ex-smokers [1,2]. However, only 3% to 5% of self-quitters achieve prolonged abstinence for 6 to 12 months after an attempt to quit [3]. Although the smoking prevalence continues to decline in Japan, according to the data of the Ministry of Health, Labour and Welfare, the rate of smokers among the entire Japanese population was still as high as 17.7% (males: 29.4%, females: 7.2%) in 2017. In Japan, patients diagnosed with nicotine dependence and those who wish to quit smoking are eligible for treatment with varenicline or nicotine patches under the National Health Insurance Program (NHIP) [4]. Under the NHIP, physicians see their patients and provide pharmacotherapy and counseling 5 times during the 12-week treatment period according to the national guidelines (The Standard Procedure Manual for Smoking Cessation, 6th edition, 2014) [5]. Although pharmacotherapy, which helps patients stop smoking, has been covered by NHIP since 2006, continuous abstinence rate (CAR) after 12 weeks has still remained low mainly because of the high dropout rate. Only 30% of the patients completed all 5 visits to see their physicians, and the average number of visits per patient was 3.3 [6]. Dropouts and the lack of visits mean that physicians cannot provide their patients sufficient support for smoking cessation. In fact, there was a negative correlation between the number of visits and the patients’ CARs [6]. It is well known that insufficient support leads to misunderstandings with regard to nicotine dependence and side effects of varenicline and nicotine patches, which eventually result in re-smoking [7-9]. Therefore, preventing dropouts and maintaining a high level of motivation and confidence to continue to remain engaged in the treatment are essential for smoking cessation therapy.

### Mobile Health and Study Objectives

Recently, mobile phone, Web-based, or smartphone apps have been considered to provide effective and continuous support for smoking cessation [10-17]. For example, Tweet2Quit, a unique social networking service–based smoking cessation Twitter program and a 12-week short message service–based intervention for university students might be effective on smoking cessation [13,14]. In addition, a smartphone smoking cessation app that offers momentary ecological assessments could be useful in providing users with timely adaptive interventions by sending various treatment messages over a 3-week period [16]. One smartphone app for smoking cessation helped 30% of its users abstain from smoking for 8 weeks [17]. However, it is yet to be known whether a smoking cessation smartphone app that provides continuous evidence-based behavioral support and personalized counseling programs improves long-term abstinence rates among nicotine-dependent patients. No official or evidence-based smoking cessation app that provides advice and scientific information and offers various alternative approaches to smokers has yet been developed in Japan.

In this research, we performed a single-arm interventional study to evaluate whether a novel smartphone app for smoking cessation, CureApp Smoking Cessation (CASC), was useful and effective for long-term abstinence in patients with nicotine dependence.

## Methods

### Study Design

This was a prospective, interventional, multicenter, single-arm study. We provided the CASC smartphone app to all the participants, and they used it daily for 24 weeks. If they wished to, participants could use this app during the entire research period (52 weeks from their registration). We asked them to keep an electronic diary regarding smoking cessation, browse messages and tutorial videos, and report withdrawal symptoms and cravings through a chat system with an artificial intelligence (AI) nurse. Participants also visited their physicians’ clinics at weeks 0, 2, 4, 8, and 12 according to the Smoking Cessation Standard Protocol in Japan [5]. Their CAR was the primary endpoint from weeks 9 to 24.

This study was approved by the Ethics Committee of Keio University School of Medicine and other facilities. Written informed consent was obtained from all the participants. This study was registered at the University Hospital Medical Information Network (UMIN) Clinical Trials Registry (UMIN000020123).

### Recruitment

We recruited adult smokers who visited the institutions for nicotine dependence treatment and were eligible for nationally insured treatment under the NHIP. Participants had to be at least 20 years of age, be diagnosed with a nicotine dependence score of greater than or equal to 5 points on the Tobacco Dependence Screener (TDS) [4], smoke cigarettes with a Brinkman index at least 200 (only for those not younger than 35 years), wish to quit smoking, own their iPhone or Android smartphone, and provide their written consent. These criteria were the same as those used to diagnose nicotine dependence under NHIP in Japan. We excluded the participants who seemed to find it difficult to use their smartphones as instructed or if their physicians ruled that they could not complete the study owing to severe mental illness.

The registration period was from March 2016 to March 2017. We followed up on the participants for 52 weeks from the time of registration. In total, 5 institutions were involved in the study, including a university hospital, 3 general hospitals, and a primary care clinic, all of which were located near the Tokyo metropolitan area.

### “CureApp Smoking Cessation” App Software

**Figure 1:**
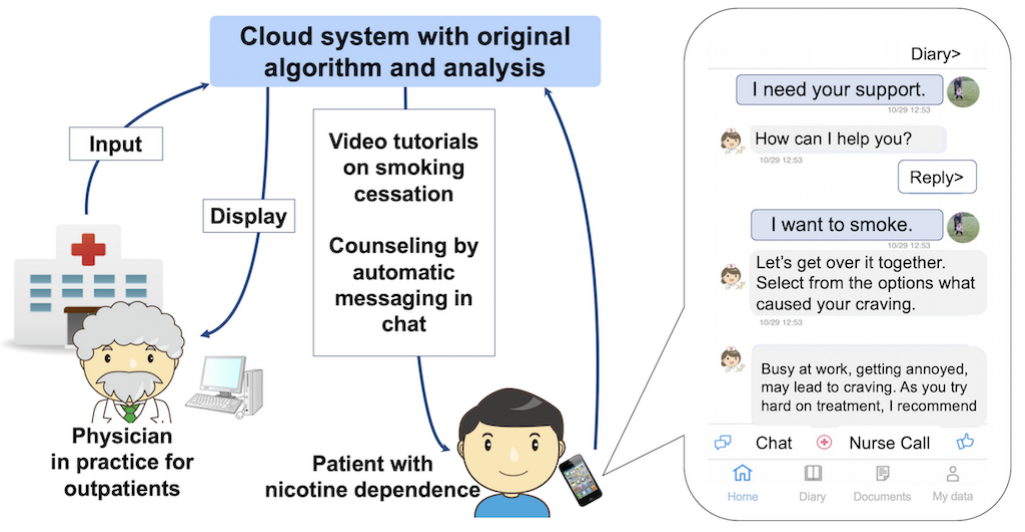
Scheme of the CureApp Smoking Cessation support program system for participants with nicotine dependence.

The CASC was developed by CureApp Inc (Tokyo, Japan) in collaboration with the Division of Pulmonary Medicine, Department of Medicine, Keio University School of Medicine. Smoking cessation specialists at the institution supervised the development of the app’s content. It was compatible with iOS and Android smartphones and met the software inspection criteria and security requirements of Apple’s App Store and Google Play. In outpatient clinics, physicians provided participants the app prescription codes. The participants downloaded the app from the App Store or Google Play using these codes, and then input their information including their age, sex, years of smoking, number of cigarettes smoked per day, medication (ie, varenicline or nicotine patch), and motivation and self-confidence regarding smoking cessation onto their smartphones. This information was securely stored on the cloud system, and our AI system created appropriate and personalized counseling advice for each participant to support their smoking cessation in accordance with the national guidelines [5]. The text of the app was presented in English for convenience (Figure 1).

Physicians could refer to the participants’ data on the website through the cloud system and offer advice to the participants during their clinic visits based on the data, as described below. The app system comprised 4 features to maximize the therapeutic effect of pharmacological therapies and counseling at outpatient clinics for smoking cessation as follows (Multimedia Appendix 1).

#### Diary of Smoking Cessation (Once a Day)

**Figure 2:**
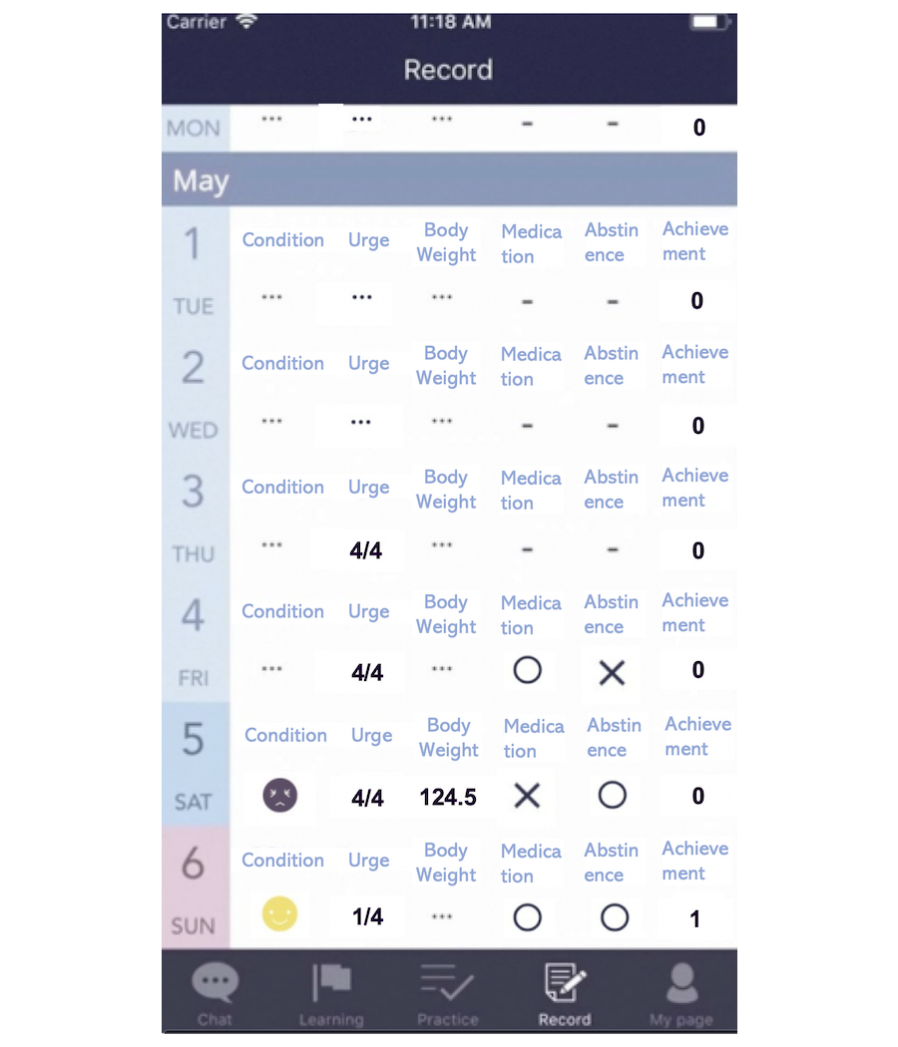
Example of the screen display for the smoking cessation diary feature.

Instead of making entries in paper diaries, participants filled in the electronic diary within this app regarding cessation status, physical condition, medication use, and adverse events, if any (Figure 2).

#### Messages and Educational Videos to Help Users Quit Smoking

**Figure 3:**
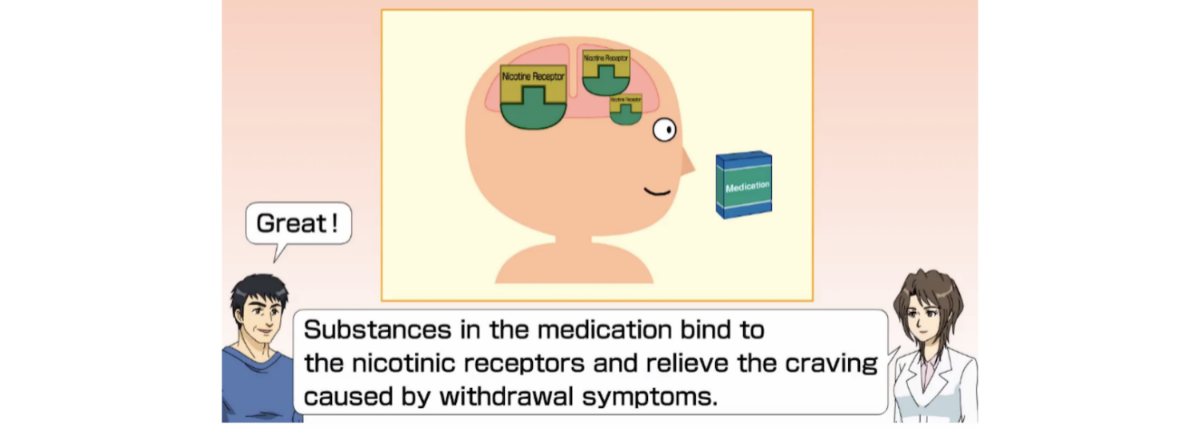
Example of the screen display for the lecture and educative videos feature.

Participants received several messages every day and watched videos (1- to 3-min animations). A total of 20 videos were delivered during the pharmacotherapy period (12 weeks). The educational videos were delivered frequently in the first few weeks, after which the frequency gradually decreased. The timing of the delivery of messages and videos varied depending on the drug prescribed (varenicline or nicotine patch) and each individual participant’s life cycle. Participants could view these videos even after week 12 or anytime they wished (Figure 3).

#### Counseling Chat Sessions Between the Users and the Artificial Intelligence Nurse

**Figure 4:**
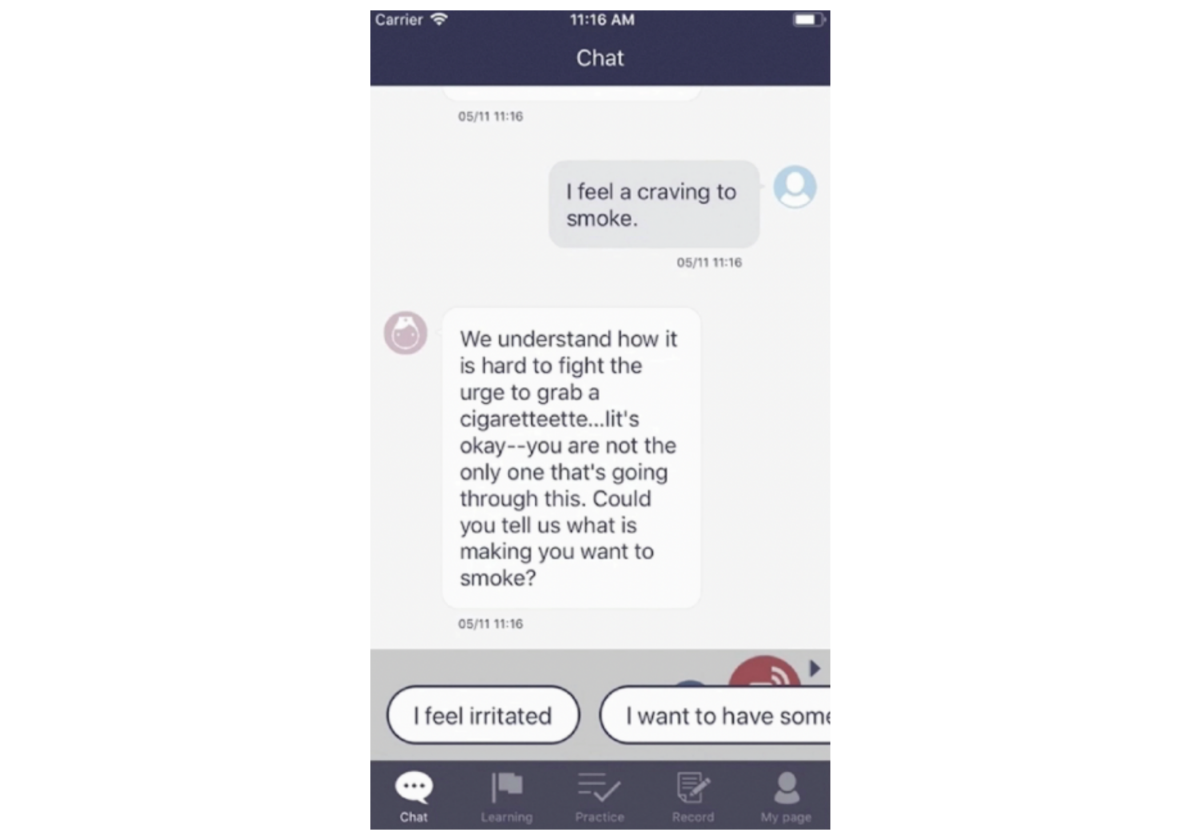
Example of the screen display for the counseling chat feature between the users and the artificial intelligence nurse.

Whenever the participants experienced cravings or withdrawal symptoms, they could tap “Call” and send a message to an AI nurse. The AI nurse would immediately reply and provide personalized advice on how to deal with the symptoms such as a chatbot. This nurse also provided encouraging messages for smoking cessation to the participants at appropriate times (Figure 4).

#### Advice for Physicians

**Figure 5:**
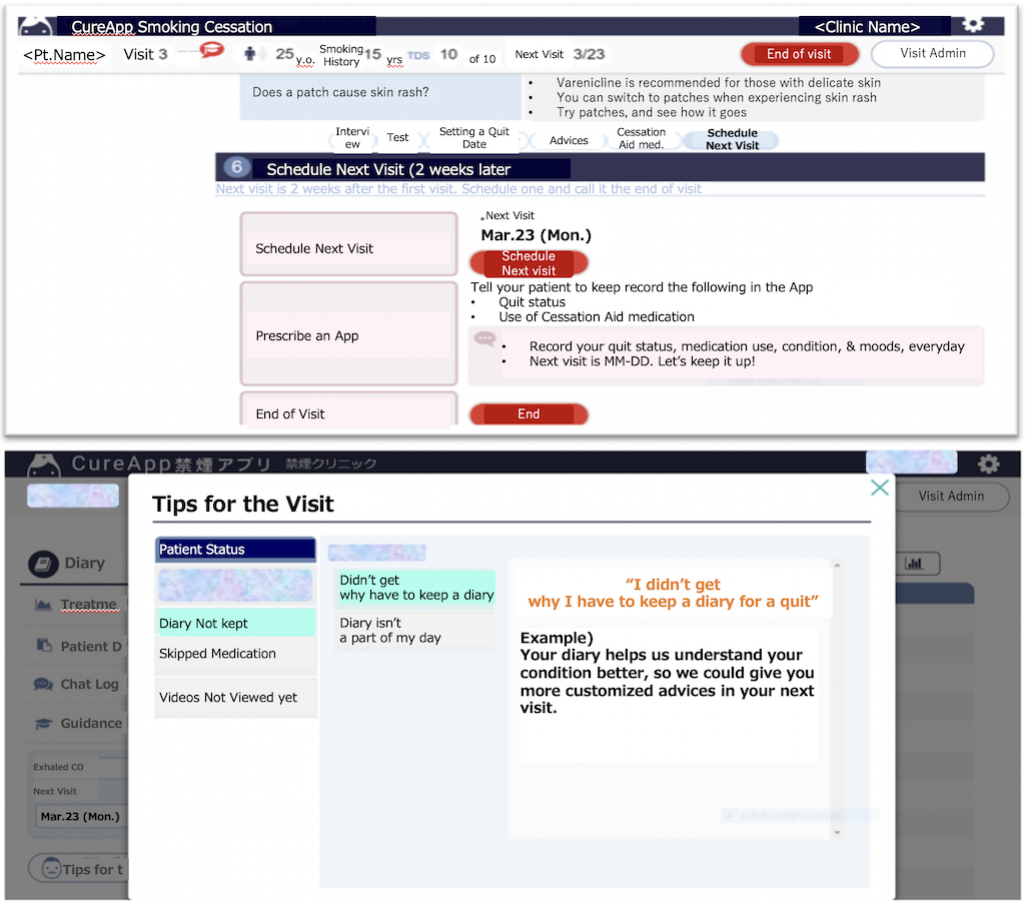
Example of the screen display feature offering advice for the physicians.

The CASC displayed recommendations to physicians on the website screen, which could be used by the physicians to offer appropriate advice and counseling support to the participants based on the national guidelines (Figure 5) [5].

### Data Collection

We collected baseline profiles of each participant including their age, sex, height, weight, Brinkman index, TDS score, and Fagerström Test for Nicotine Dependence (FTND) score [18] on their first visit. Smoking status, medication use (varenicline or nicotine patch), exhaled carbon monoxide (CO) concentration, as well as their scores on the Mood and Physical Symptoms Scale (MPSS) [19], 12-item French version of the Tobacco Craving Questionnaire (FTCQ-12) [20], and Kano Test for Social Nicotine Dependence (KTSND) [21] were collected at every visit (weeks 2, 4, 8, and 12). The MPSS is a validated questionnaire that assesses the severity of participants’ withdrawal symptoms and the strength and frequency of their urges to smoke, with scores ranging from 5 to 35. The FTCQ-12 is a valid 12-item self-report instrument that assesses tobacco cravings based on 4 factors: emotionality, expectancy, compulsivity, and purposefulness. The FTCQ-12 general craving score is derived by summing all items and dividing the total by 12, yielding a score ranging from 1 to 7. The KTSND is a method of evaluating the psychological aspects of smoking and comprises 10 questions with a total score range of 0 to 30. In these 3 scoring systems, higher scores indicate more severe symptoms and features. We also gathered the app usage status such as the number of days the participants updated their diaries, the number of behaviors they actually modified as a result of counseling, and the number of educational videos they viewed completely. The primary endpoint was the CAR from weeks 9 to 24. Secondary endpoints were CARs from weeks 9 to 12 and 9 to 52 and changes in MPSS, FTCQ-12, and KTSND scores.

### Confirmation of Continuous Smoking Cessation

Self-reported continuous abstinence was confirmed if participants recorded a breath CO concentration of less than 8 ppm during their clinic visits until week 12. Confirmation of abstinence at weeks 24 and 52 was determined by face-to-face or telephone interviews.

### Statistical Analysis

We analyzed the primary and secondary endpoints based on the full analysis set (FAS) and compared our data with those of the national survey of Japan [22]. CI for the binomial proportion was computed using the Agresti-Coull method.

## Results

### Recruitment and Baseline Characteristics

**Figure 6:**
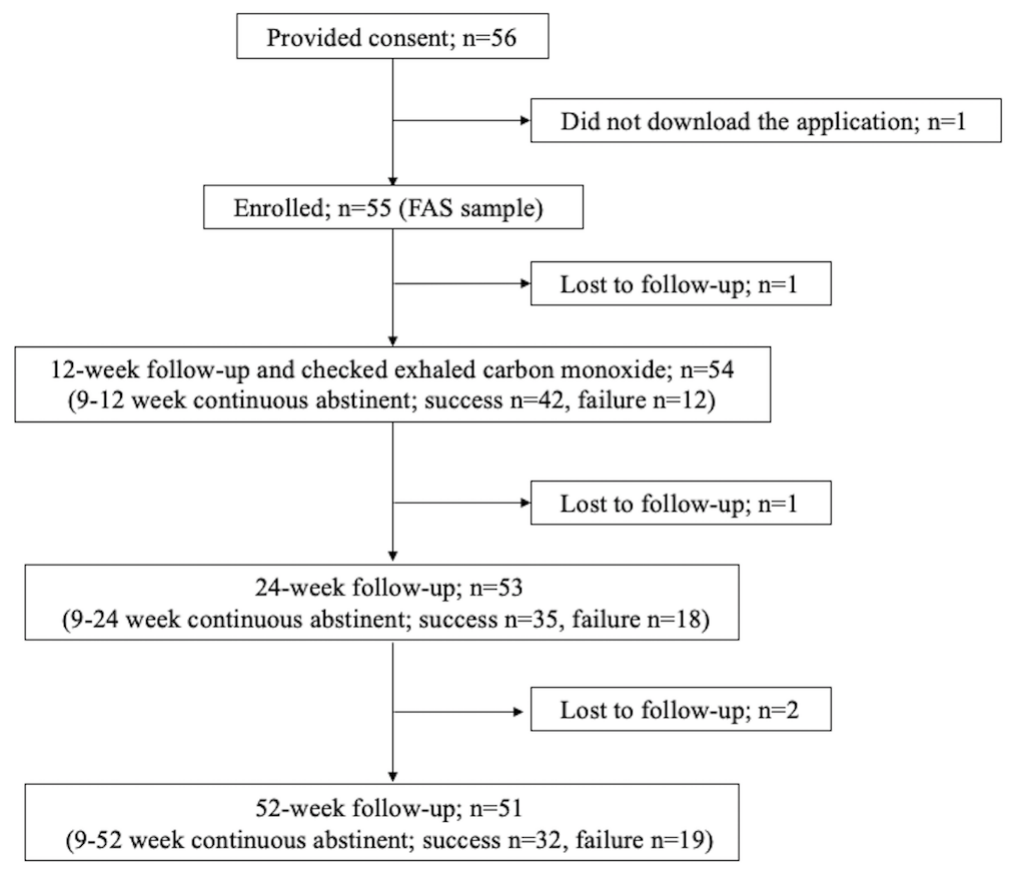
Flowchart depicting the selection of study participants.

We recruited 56 participants with nicotine dependence who met the inclusion criteria. Of these participants, because 1 did not download the app, the FAS sample comprised 55 participants. From this FAS sample, 1 participant dropped out during weeks 0 to 12, 1 dropped out during weeks 12 to 24, and 2 dropped out during weeks 24 to 52. These dropouts were treated as failures. A flowchart of the study is depicted in Figure 6.

The baseline characteristics of the study participants are depicted in Table 1. The participants’ mean age was 43.3 years and 71% of them were male. Some of the study participants had comorbidities, such as chronic obstructive pulmonary disease (13%), hypertension (7%), cancer (7%), diabetes mellitus (5%), and dyslipidemia (5%), and these comorbidities were all under control and in stable conditions. The mean of Brinkman index was 486, baseline exhaled CO concentration was 20.6 ppm, and TDS was 7.9. Varenicline was administered to 87% (48/55) of the participants, and 13% (7/55) received nicotine patches. Of the participants, 1 used both varenicline and a nicotine patch sequentially, and another participant used neither varenicline nor a nicotine patch. The mean FTND, the MPSS total score, the FTCQ-12 general craving score, and the KTSND score at the first visit were 5.5, 20.6, 3.2, and 16.6, respectively.

### Evaluation Outcomes

Of the FAS sample, 35 participants succeeded in continuously abstaining from smoking until the 24th week. The CAR from weeks 9 to 24 was 64% (35/55, 95% CI 51%-76%); see Table 2). The CARs from weeks 9 to 12 and 9 to 52 were 76% (42/55, 95% CI 65%-88%) and 58% (32/55, 95% CI 46%-71%), respectively.

The total score of the MPSS at week 12 was 13.7, which decreased by 6.4 points from the baseline. The mean MPSS scores related to “depressed,” “irritable,” “restless,” “hungry,” and “poor concentration” at week 12 were 1.3, 1.4, 1.3, 2.2, and 1.7, respectively. Similarly, the mean score of the FTCQ-12 (general craving score) at week 12 was 2.5, which had reduced by 0.6 points from the baseline. The mean FTCQ-12 scores related to “emotionality” and “compulsivity” at week 12 were 1.2 and 1.6, respectively. They had decreased from the baseline scores. On the other hand, the mean FTCQ-12 score related to “expectancy” barely changed from baseline, whereas the mean FTCQ-12 score related to “purposefulness” at week 12 was 4.7 on average and had increased from the baseline. The KTSND score at week 12 was 10.5 and had decreased by 6.7 points from the baseline.

### App Usage

There were several indicators of the app’s usage status (Table 3). On average, the participants tapped “Like!” 26.5 times from weeks 0 to 12, which implied that they agreed with the advice provided by the AI nurse. They tapped “Call,” which summoned the AI nurse when participants had smoking impulses or side effects, an average of 1.7 times from weeks 0 to 12. They mean number of days on which participants made diary entries from weeks 0 to 12 was 56.1, and 45.5% (25/55) of the participants updated their diaries every day. The mean number of educational videos viewed from start to finish by the participants was 12.6.

### Adverse Events

In total, 3 participants, all of whom took varenicline reported 3 adverse events. Skin eruptions appeared in 1 participant and mild nausea appeared in another. Both of these participants could continue to take varenicline. Depressive symptoms appeared in 1 participant in the 9th week, and the physician decided to stop the varenicline treatment for this participant. On the basis of their judgment, the physicians concluded that these adverse events were caused by the drugs rather than by the app.

**Table 1:** Baseline characteristics of study participants (N=55).

Participant characteristics	Statistics
Age (years), mean (SD)	43.3 (10.5)
Males, n (%)	39 (71)
Body weight (kg), mean (SD)	63.1 (10.7)
Body mass index (kg/cm^2^), mean (SD)	23.3 (3.4)
Years of smoking, mean (SD)	23.6 (10.8)
Number of cigarettes smoked per day, mean (SD)	19.9 (7.3)
Brinkman index, mean (SD)	486 (329)
Number of lifelong cessation attempts, mean (SD)	1.1 (1.2)
Exhaled CO^a^ concentration (ppm), mean (SD)	20.6 (15.9)
FTND^b^, mean (SD)	5.5 (1.9)
TDS^c^, mean (SD)	7.9 (1.4)
KTSND^d^, mean (SD)	16.6 (4.3)
MPSS^e^ total, mean (SD)	20.6 (4.7)
FTCQ-12^f^ general craving score, mean (SD)	3.2 (0.7)
Varenicline, n (%)	48 (87)
Nicotine patch, n (%)	7 (13)
No pharmacotherapy, n (%)	1 (2)

^a^CO: carbon monoxide.

^b^FTND: Fagerström Test for Nicotine Dependence.

^c^TDS: Tobacco Dependence Screener.

^d^KTSND: Kano Test for Social Nicotine Dependence.

^e^MPSS: Mood and Physical Symptoms Scale.

^f^FTCQ-12: 12-item French version of the Tobacco Craving Questionnaire.

**Table 2:** Primary and secondary endpoints.

Endpoints	Statistics
CAR^a^ from weeks 9 to 12, % (95% CI)	76 (65-88)
CAR from weeks 9 to 24 (primary endpoint), % (95% CI)	64 (51-76)
CAR from weeks 9 to 52, % (95% CI)	58 (46-71)
MPSS^b^ total at week 12, mean (SD)	13.7 (3.5)
ΔMPSS total from baseline to week 12, mean (SD)	–6.4 (5.8)
FTCQ-12^c^ general craving score at week 12, mean (SD)	2.5 (1.1)
ΔFTCQ-12 general craving score from baseline to week 12, mean (SD)	–0.6 (1.5)
KTSND^d^ at week 12, mean (SD)	10.5 (5.9)
ΔKTSND from baseline to week 12, mean (SD)	–6.7 (5.2)

^a^CAR: continuous abstinence rate.

^b^MPSS: Mood and Physical Symptoms Scale.

^c^FTCQ-12: 12-item French version of the Tobacco Craving Questionnaire.

^d^KTSND: Kano Test for Social Nicotine Dependence.

**Table 3:** App usage from weeks 0 to 12.

Engagement with the app	Mean (SD)
Number of times “Like!” was tapped	26.5 (63.8)
Number of times “Call” was tapped	1.7 (2.4)
Number of days a diary entry was made	56.1 (31.3)
Number of educational videos viewed	12.6 (6.8)
Practices for environmental improvement	67.5 (135.6)
Behaviors actually modified	74.3 (158.5)
Practices for compensatory behaviors	97.4 (374.3)
Practices for self-assertiveness	8.2 (17.3)

## Discussion

### Principal Findings

This was the first prospective study to evaluate whether a novel smartphone app, CASC, could achieve long-term CARs from weeks 9 to 24 in nicotine-dependent patients. Our results showed that more than three-fifths of participants succeeded in quitting smoking at week 24. The combination of CASC and conventional pharmacotherapy resulted in more than three-fourths of all participants maintaining their CARs from weeks 9 to 12, and 76% (32/42) of quitters at week 12 continued to abstain from smoking at week 52. This combination treatment also decreased the participants’ MPSS, FTCQ-12 (general craving), and KTSND scores by week 12 of the study period. Except 1 participant who dropped out during the 12 weeks of the treatment period, all others remained, and many participants tapped “Like!” in response to the advice they received from the AI nurse and updated the diary daily, indicating that the usage of CASC was feasible.

**Figure 7:**
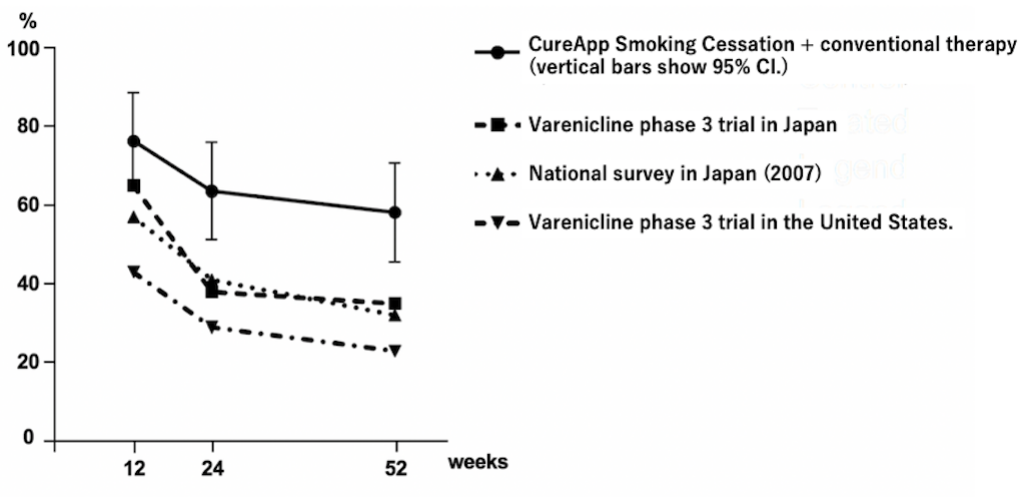
Continuous abstinence rates from weeks 9 to 12, 9 to 24, and 9 to 52.

The CAR from weeks 9 to 24 obtained in this study appeared to be substantially better than those of previous studies and of the national survey (Figure 7) [22-24]. According to the national survey data and the varenicline phase 3 trial in Japan, the CAR from weeks 9 to 24 were 40.81% (1039/2546) and 37.7% (49/130), respectively [22,23]. The CARs from weeks 9 to 12, 9 to 24, and 9 to 52 were 76% (42/55, 95% CI 65%-88%), 64% (35/55, 95% CI 51%-76%), and 58% (32/55, 95% CI 46%-71%), respectively.

There are several reasons why our smartphone app could improve long-term CARs. First, it was designed to promote understanding of nicotine dependence through several evidence-based approaches. For example, educational videos included the following treatment programs: education about nicotine dependence, explanation of nicotine withdrawal symptoms, and behavioral therapy. Consequently, the quitters at week 12 did not relapse frequently because they might have understood that smoking behavior was caused by the dependence on nicotine and because they were aware of the characteristics of addictive behaviors. In fact, the KTSND score, which was considered as one of the indices reflecting the understanding of nicotine dependence [21], decreased more than 6 points from weeks 0 to 12 (Table 2).

Second, continuous encouragement and timely advice by an AI nurse might prevent dropouts during the treatment. All but 1 participant (92%) completed the outpatient smoking cessation treatment for 12 weeks in our study, whereas only 30% of the participants completed the treatment that was a part of the domestic survey data [6]. Notably, the existence of an AI nurse might keep participants from dropping out because many studies have shown that smoking cessation success rates improve with interventions not only by physicians but also by nurses and pharmacists [25-30]. In a single fight, patients easily abandon smoking cessation because of cravings and withdrawal symptoms. Besides, maintaining one’s motivation to make multiple attempts considering several aspects was reported to be important in smoking cessation [31]. These studies indicated the importance of encouragement and support during the pharmacotherapy period. Therefore, individualized support and encouragement for each patient is essential for an app to maximize the effect of the medication and counseling. The AI nurse advised the participants of this study about how to deal with cravings for smoking as if it were an expert partner who was accompanying them while they were trying to quit smoking. In this study, not only the physician but also the CASC may have functioned as a supporter of and advisor to participants.

Third, adequate advice through educational videos to prevent relapse might help the quitters continue to abstain from smoking easily. In fact, the MPSS total score and FTCQ-12 general craving score decreased from baseline to week 12 (Table 2). The decrease in the urge to smoke might contribute to the continuation of smoking cessation from weeks 12 to 24 and 52.

Finally, the app might prevent treatment failure because of the adverse effects of the varenicline or nicotine patch. It was decided that only 1 participant would stop varenicline treatment owing to its adverse effects. On our app, the alarm feature reminding the participants to take their medicines and an explanation of the side effects were different for participants who received varenicline and for those who received the nicotine patch. Participants could learn how to deal with the adverse effects by watching the educational videos and reading the messages sent by the AI nurse.

### Strengths and Limitations

The strengths of this study are as follows: (1) this study is the first to measure the long-term effectiveness and feasibility of smartphone app intervention for more than 24 weeks; (2) the features of our app were designed based on the national treatment guidelines [5], and it was different from other apps that aimed merely to record the participants’ trajectories; and (3) our results also highlighted the additional effect of the app on pharmacotherapy. The limitations of this study are as follows: (1) because the sample size was small, the study participants might not have represented all Japanese smokers; (2) participating institutes were restricted only to the area around Tokyo in Japan; (3) the education level of the study participants was not surveyed, which might have influenced the results; and (4) we did not confirm abstinence with exhaled CO concentration at weeks 24 and 52. In previous intervention studies, confirmation of self-assessment through telephone surveys was a standard when the study design was minimally invasive [32]. In our study, all 35 successful quitters at week 24 had shown normal exhaled CO levels during their week 12 visits, indicating the reliability of the self-reports in our sample.

### Conclusions

In conclusion, a novel smartphone app for smoking cessation, CACS, benefitted 63.6% of the users who maintained their CARs from weeks 9 to 24. Our 95% CI ranges of CARs were superior to those of the historical cohorts [22-24] (Figure 7). This app might be a useful tool for improving long-term CAR combined with a standard smoking cessation treatment program. Currently, a randomized placebo app–controlled trial is ongoing to evaluate the efficacy of the app for nicotine dependence (UMIN000031589). We will continue to improve this app further to make it an available and essential tool in smoking cessation therapy.

## Appendix

Multimedia Appendix 1 How the app appears on a patient's smartphone screen.

## Abbreviations

AI: artificial intelligence
CAR: continuous abstinence rate
CASC: CureApp Smoking Cessation
CO: carbon monoxide
FAS: full analysis set
FTCQ-12: 12-item French version of the Tobacco Craving Questionnaire
FTND: Fagerström Test for Nicotine Dependence
KTSND: Kano Test for Social Nicotine Dependence
MPSS: Mood and Physical Symptoms Scale
NHIP: National Health Insurance Program
TDS: Tobacco Dependence Screener
UMIN: University Hospital Medical Information Network
